# Supplementary material for: Identification of Urine Metabolic Markers of Stroke Risk Using Untargeted Nuclear Magnetic Resonance Analysis
Source: Int J Mol Sci. 2024 Jul 6;25(13):7436. doi: 10.3390/ijms25137436 (PMC11242327; doi:10.3390/ijms25137436)
Supplement: Supplementary file 1 [file ijms-25-07436-s001.zip › ijms-3029450_Table S1.pdf]

Table S1 – Assignments of chemical shifts of metabolites from 600MHz ^{1}H-NMR NOESY urine pool spectra. Peaks multiplicities are represented by singlet (s), doublet (d), triplet (t), multiplet (m) and double doublet (dd).

Pool spectra number	Compound	Chemical shift (δ ^{1}H ppm)	Multiplicity (*)
1	TSP	0.00	s
2	Isoleucine	1.00	d
3	Valine	1.05	q
4	Isobutyrate	1.07	d
5	Methylsuccinate	1.11	m
6	Ethanol	1.19	t
7	3-hydroxyisovalerate (3-HIVA)	1.27	s
8	Threonine	1.34	d
9	Alpha-hydroxyisobutyrate (2-HIBA)	1.36	s
10	Alanine	1.49	q
11	Lysine	1.90	m
12	Acetate	1.92	s
13	2-hydroxyglutarate	1.99	m
14	N-acetylaspartate	2.04	s
15	N-acetylneuraminate	2.07	s
16	Acetone	2.17	s
17	Acetoacetate	2.18	s
18	Glutamate	2.28	m
19	Pyruvate	2.35	s
20	Succinate	2.41	t
21	Glutamine	2.44	m
22	Citrate	2.61	dd
23	Dimethylamine	2.72	s
24	Creatine	3.04	s
25	Creatinine	3.04; 4.06	s; s
26	Methylurate	3.16	s
27	Trimethylamine N-oxide (TMAO)	3.26	s
28	Taurine	3.27; 3.42	t, t
29	Methanol	3.36	s
30	4-hydroxyphenylacetate (4-HPA)	3.44; 7.15	s; d
31	Total sugar (monosaccharides, disaccharides)	3.53; 3.75	m, m
32	Glycine	3.57	s
33	Glycolate	3.94	s
34	Serine	3.95	m
35	Hippurate	3.97; 7.56; 7.64; 7.84	d; m; t; d
36	Lactate	4.11	q
37	Trigonelline	4.44; 8.09; 8.84; 9.12	s; t; m; s
38	Urea	5.81	s
39	Histidine	7.10	s
40	Indoxyl sulphate	7.21; 7.30	dd; dd
41	Phenylacetylglutamine (PAG)	7.36	m

42	Phenylalanine	7.44	m
43	Nudifloramide	8.34	d
44	Formate	8.47	s
