# Supplementary material for: Identification of Urine Metabolic Markers of Stroke Risk Using Untargeted Nuclear Magnetic Resonance Analysis
Source: Int J Mol Sci. 2024 Jul 6;25(13):7436. doi: 10.3390/ijms25137436 (PMC11242327; doi:10.3390/ijms25137436)
Supplement: Supplementary file 1 [file ijms-25-07436-s001.zip › ijms-3029450_Table S2.pdf]

Table S2 – Absolute concentrations (mmol/L & mg/dL) of the 44 metabolites/signals identified in urine samples from elderly people belonging to the study groups, namely low stroke risk (LSR, control, n=73), moderate stroke risk (MSR, n=87), and high stroke risk (HSR, n=17). Data are expressed as "Mean ± s.e.m"." Statistical significance was analyzed with non-parametric Kruskal-Wallis test (KWT) and One-way ANOVA test (OWAT) depending on the homogeneity of variance of the data (*P < 0.05).

Metabolites	Low	Moderate	High	P-value	Statistical test
Isoleucine (mmol/L)	0.270 ± 0.018	0.215 ± 0.011	0.301 ± 0.048	0.078	KWT
Valine (mmol/L)	0.214 ± 0.015	0.190 ± 0.012	0.207 ± 0.035	0.464	OWAT
Isobutyrate (mmol/L)	0.224 ± 0.018	0.201 ± 0.012	0.245 ± 0.038	0.705	KWT
Methylsuccinate (mmol/L)	0.442 ± 0.030	0.371 ± 0.0209	0.470 ± 0.067	0.145	KWT
Ethanol (mmol/L)	1.383 ± 0.117	1. 445 ± 0.129	1. 486 ± 0.268	0.913	OWAT
3-hydroxyisovalerate (3-HIVA) (mmol/L)	0.385 ± 0,030	0.295 ± 0.015	0.394 ± 0.069	0.249	KWT
Threonine (mmol/L)	1.580 ± 0.140	1.672 ± 0.148	1.479 ± 0.280	0.815	OWAT
Alpha-hydroxyisobutyrate (2-HIBA) (mmol/L)	0.568 ± 0.039	0.496 ± 0.023	0.655 ± 0.097	0.455	KWT
Alanine (mmol/L)	1.903 ± 0.0147	1.631 ± 0.112	1.793 ± 0.291	0.478	KWT
Lysine (mmol/L)	2.103 ± 0.142	1.854 ± 0.122	1.832 ± 0.232	0.241	KWT
Acetate (mmol/L)	2.267 ± 0.340	2.941 ± 0.461	0.838 ± 0.129	0.096	KWT
2-hydroxyglutarate (mmol/L)	3.243 ± 0.186	2.962 ± 0.131	3.737 ± 0.424	0.237	KWT
N-acetylaspartate (mmol/L)	1.733 ± 0.101	1.836 ± 0.98	2.235 ± 0.393	0.552	KWT
N-acetylneuraminate (mmol/L)	2.331 ± 0.172	2.041 ± 0.103	2.665 ± 0.392	0.490	KWT
Acetone (mmol/L)	0.283 ± 0.050	0.401 ± 0.114	0.974 ± 0.457	0.214	KWT
Acetoacetate (mmol/L)	0.272 ± 0.050	0.311 ± 0.098	0.552 ± 0.268	0.421	OWAT
Glutamate (mmol/L)	20.829 ± 1.516	19.305 ± 1.320	16.952 ± 2.117	0.462	OWAT
Pyruvate (mmol/L)	4.478 ± 0.388	4.528 ± 0.361	3.367 ± 0.582	0.394	OWAT
Succinate (mmol/L)	0.759 ± 0.057	0.849 ± 0.165	0.849 ± 0.165	0.762	OWAT
Glutamine (mmol/L)	2.852 ± 0.200	2.400 ± 0.135	2.861 ± 0.493	0.403	KWT
Citrate (mmol/L)	22.880 ± 2.212	18.920 ± 1.495	22.664 ± 5.892	0.713	KWT
Dimethylamine (mmol/L)	2.330 ± 0.143	2.177 ± 0.109	2.717 ± 0.388	0.516	KWT
Creatine (mmol/L)	0.006 ± 0.001	0.008 ± 0.001	0.006 ± 0.003	0.253	OWAT
Creatinine (mmol/L)	68.297 ± 4.541	57.634 ± 2.738	75.586 ± 12.374	0.382	KWT
Methylurate (mmol/L)	0.737 ± 0.567	0.644 ± 0.036	0.738 ± 0.100	0.641	KWT
Trimethylamine N-Oxide (TMAO) (mmol/L)	0.038 ± 0.007	0.023 ± 0.002	0.039 ± 0.006	0.042*	KWT
Taurine (mmol/L)	22.089 ± 1.986	22.610 ± 3.439	21.485 ± 1.378	0.872	OWAT
Methanol (mmol/L)	0.475 ± 0.036	0.415 ± 0.264	0.557 ± 0.081	0.119	OWAT
Total sugar (monosaccharides, disaccharides) (mg/dL)	96.720 ± 7.298	72.910 ± 3.907	92.958 ± 10.113	0.050*	KWT
Glycine (mmol/L)	4.022 ± 0.408	3.835 ± 0.328	2.822 ± 0.632	0.381	OWAT
Glycolate (mmol/L)	1.337 ± 0.165	1.636 ± 0.220	0.672 ± 0.138	0.050*	KWT
Serine (mmol/L)	2.657 ± 0.204	2.418 ± 0.125	3.254 ± 0.434	0.245	KWT
Lactate (mmol/L)	4.652 ± 0.296	4.373 ± 0.232	5.944 ± 0.926	0.403	KWT
Urea (mmol/L)	20.574 ± 1.204	17.666 ± 0.749	22.264 ± 2.736	0.178	KWT
Histidine (mmol/L)	0.981 ± 0.093	0.689 ± 0.051	0.997 ± 0.215	0.130	KWT

4-hidroxyphenylacetate (4-HPA) (mmol/L)	0.605 ± 0.087	0.659 ± 0.147	2.071 ± 0.721	0.018*	KWT
Indoxyl sulphate (mmol/L)	11.197 ± 0.821	10.199 ± 0.663	10.127 ± 1.236	0.592	OWAT
Phenylacetylglutamine (mmol/L)	4.106 ± 0.311	3.620 ± 0.245	3.691 ± 0.401	0.654	OWAT
Phenylalanine (mmol/L)	4.106 ± 0.311	3.620 ± 0.245	3.691 ± 0.401	0.430	OWAT
Hippurate (mmol/L)	8.970 ± 0.949	6.694 ± 0.648	6.521 ± 1.310	0.368	KWT
Nudifloramide (mmol/L)	0.582 ± 0.039	0.607 ± 0.040	0.735 ± 0.137	0.782	KWT
Formate (mmol/L)	1.011 ± 0.110	0.689 ± 0.070	0.663 ± 0.128	0.075	KWT
Trigonelline (mmol/L)	0.799 ± 0.076	0.505 ± 0.033	0.434 ± 0.042	0.050*	KWT
